# Is There Really a Difference in Outcomes between Men and Women with Hepatocellular Cancer?

**DOI:** 10.3390/cancers15112892

**Published:** 2023-05-24

**Authors:** Andrea Fa, Denise M. Danos, Lauren Maniscalco, Yong Yi, Xiao-Cheng Wu, Mary A. Maluccio, Quyen D. Chu, John M. Lyons

**Affiliations:** 1 School of Medicine, LSU Health Sciences Center-New Orleans, New Orleans, LA 70112, USA; afa@lsuhsc.edu (A.F.);; 2 School of Public Health, LSU Health Science Center-New Orleans, New Orleans, LA 70112, USA; 3 Louisiana Tumor Registry, School of Public Health, LSU Health Science Center-New Orleans, New Orleans, LA 70112, USA; 4 Orlando Health Cancer Institute, Orlando, FL 32806, USA; 5 Our Lady of the Lake Regional Medical Center, Baton Rouge, LA 70808, USA

**Keywords:** hepatocellular cancer, Louisiana, incidence, women, disparities

## Abstract

**Simple Summary:**

Hepatocellular carcinoma has a clear male predominance, but gender-specific differences remain incompletely understood. Our findings look specifically at the Louisiana population, which has some of the highest rates of hepatitis, alcoholic liver disease, and metabolic syndrome—thus providing important data to better evaluate HCC trends. Our findings help illustrate specific gender differences in this disease, which remains one of the leading causes of cancer-related deaths worldwide.

**Abstract:**

Hepatocellular carcinoma (HCC) is a male-dominated disease. Currently, gender differences remain incompletely defined. Data from the state tumor registry were used to investigate differences in demographics, comorbidities, treatment patterns, and cancer-specific survival (HSS) among HCC patients according to gender. Additional analyses were performed to evaluate racial differences among women with HCC. 2627 patients with HCC were included; 498 (19%) were women. Women were mostly white (58%) or African American (39%)—only 3.8% were of another or unknown race. Women were older (65.1 vs. 61.3 years), more obese (33.7% vs. 24.2%), and diagnosed at an earlier stage (31.7% vs. 28.4%) than men. Women had a lower incidence of liver associated comorbidities (36.1% vs. 43%), and more often underwent liver-directed surgery (LDS; 27.5% vs. 22%). When controlling for LDS, no survival differences were observed between genders. African American women had similar HSS rates compared to white women (HR 1.14 (0.91,1.41), *p* = 0.239) despite having different residential and treatment geographical distributions. African American race and age >65 were predictive for worse HSS in men, but not in women. Overall, women with HCC undergo more treatment options—likely because of the earlier stage of the cancer and/or less severe underlying liver disease. However, when controlling for similar stages and treatments, HCC treatment outcomes were similar between men and women. African American race did not appear to influence outcomes among women with HCC as it did in men.

## 1. Introduction

Hepatocellular carcinoma (HCC) is the fourth leading cause of cancer death worldwide [1]. In the United States, the American Cancer Society estimated 41,260 new cases of primary liver and bile duct cancer in 2022 alone. Despite public health efforts, HCC remains one of the few malignancies that continues to increase in both incidence and in deaths [2].

HCC predominantly affects males, with an incidence that is two to four-times more common than that in women [3]. The reasons for this are complex, multifactorial, and incompletely understood. Men have a higher incidence of viral hepatitis and suffer more frequently from alcoholic liver disease [4,5,6]. Additionally, the higher concentration of estrogen found in women has been shown to reduce hepatic inflammation and is theorized to be protective against the malignant transformation of hepatocytes in females [7,8,9].

The incidence of obesity in the United States is higher amongst women than men [10], and obesity has been shown to be a risk factor for HCC development [11,12]. As fat-related liver diseases emerge as the most common etiology of chronic liver disease (over viral hepatitis) [13], it is highly possible that the gender demographics classically associated with HCC may change.

The state of Louisiana harbors a large population of minority and impoverished citizens and consequently ranks fifth in HCC incidence [14] and second in HCC mortality [15] within the United States. Thus, studying this disease in Louisiana has unique implications that are not reproducible in large tertiary care centers nor in population registries. It is critical to identify gender-specific strategies to improve cancer detection and management in this vulnerable group of patients. The aim of this study was to comprehensively evaluate a large cohort of HCC patients to better identify potential inherent risk factors associated with gender and to compare treatment patterns and outcomes.

## 2. Materials and Methods

### 2.1. Data Source and Study Population

Study data was derived from the Louisiana Tumor Registry (LTR), which is a population-based registry funded by the NCI’s SEER Program and the Centers for Disease Control and Prevention’s National Program of Cancer Registries (NCPR) [16]. This registry collects and adjudicates cancer incidence data for 100% of the state’s population to support cancer prevention and control research activities [17]. Data from Our Lady of the Lake and Louisiana State University were included in this database. Primary invasive HCC cases diagnosed in individuals aged 20 and older between the years 2005 and 2015 were identified using the ICD-O-3 topographic code C220 and histology codes 8170–8180. This study excluded cases diagnosed at death or cases without follow-up duration. The study was exempted from Institutional Review Board (IRB) approval by the Louisiana State University Health Sciences Center, New Orleans.

### 2.2. Case Characteristics

Variables pertaining to demographics and patient characteristics were standard North American Association of Central Cancer Registries (NAACCR) items listed in the registry. Liver-associated comorbidities (LAC) were defined using the mild and moderate to severe liver disease categories of the NCI’s comorbidity index, using ICD-9 diagnosis codes listed within the registry [18]. Consequently, LAC included conditions related to viral hepatitis, liver necrosis, chronic liver disease and cirrhosis, and liver abscesses and sequelae of chronic liver disease. The Charlson Comorbidity Index was also calculated using ICD-9 diagnosis codes in the registry [19]. BMI was calculated according to the standard formula (BMI = weight (kilograms) divided by height squared (meters)) when the patient’s height and weight was given [20]. BMI was categorized as lean (BMI < 30) and obese (BMI ≥ 30). BMI was listed as a descriptive variable only, as the capturing of weight and height data by the LTR only started after 2010. AJCC Stage was defined by pathologic stage, or clinical stage if pathological stage was not recorded. Surgery status was determined using NAACCR surgery summary codes and classified as no surgery of primary site, ablative type procedures, surgical resections, and transplantation. When code surgery summary was not otherwise specified, it was attributed to resection for the purpose of this analysis. Non-surgical treatments, including radiologic arterial-based treatments, were not included in this dataset; therefore, they were not included as liver-directed treatments in this report.

Residential rural–urban status was based on address at time of diagnosis and classified according to the US Department of Agriculture Rural Urban Continuum codes [21]. The top 5 parishes (counties) in the state treated at least 225 patients over the study period, which represents the top 15% of parishes. Thus, parish case volume was categorized as low or high volume using a cutoff of 225 patients over the course of the study period.

### 2.3. Survival Outcomes

The primary outcome of HCC-specific survival (HSS) was defined as days from date of diagnosis to cancer-specific death (underlying cause of death was HCC) or date of last contact. Survival analyses excluded cases in which HCC was not the first primary cancer.

### 2.4. Statistical Analysis

Group comparisons of demographic, staging, treatment, and outcome coordinate variables between these two groups were analyzed using ANOVA for normally distributed continuous measures, and Chi-squared tests. Fisher’s exact test was used in the instance of small cell counts (<5). This study excluded cases with missing treatment or facility attributes. The median HSS was estimated using Kaplan–Meier estimators and compared via log-rank tests. Multivariable Cox proportional hazards models were used to evaluate trends and associations regarding HSS. Cases with an unknown AJCC stage were excluded from the Cox proportional hazards model. Model 1 included age, sex, race, liver disease, stage, and parish case volume as fixed effects. Model 2 included the same covariates and also controlled for liver-directed surgery (LDS). Categorical comparisons and survival models were executed in SAS/STAT software, version 9.4 (Cary, NC, USA).

## 3. Results

### 3.1. Patient Characteristics

A total of 2627 patients were included in the analysis. Patient characteristics are outlined in Table 1—overall and by gender. The mean age of the HCC patients was 62.1 years (range 24–96). Of 2627 patients, 19% were women (*n* = 498) and 81% were men (*n* = 2129). The majority of patients were White (58%) or Black (39%), while 3.8% were of another or unknown race. Only 52% (*n* = 1379) of patients had a documented AJCC stage. Of these patients, 473 (34.3%) had stage I disease, 289 (21%) had stage II, 313 (22.7%) had stage III, and 304 (22%) had stage IV. The majority of cases (86.7%) lived in a metropolitan core area; 1081 (41.2%) had one or more liver-associated comorbidities reported to the registry. Most cases (52%) had a Charlson Comorbidity Index (CCI) score of two, while 110 (4.2%) had a CCI score of three, 710 (27.0%) had a CCI score of four, and 446 (17%) had a CCI score of five or greater. Of the 1220 patients with recorded BMI data, the average BMI was 27.2 and the standard deviation was 6.02; 905 (74.2%) patients were categorized as lean and 315 (25.8%) patients were obese.

Of the 2627 HCC patients in the study, 2021 (76.9%) did not undergo liver-directed surgery; 105 (4%) patients underwent ablative treatment, 276 (10.5%) patients underwent liver resection, and 225 (8.6%) patients underwent liver transplantation. The median time from diagnosis to time of treatment of HCC was 27 days—54.1% patients had treatment within 30 days of diagnosis and 67.6% had treatment within 45 days of diagnosis.

### 3.2. Gender Characteristics: Men vs. Women

The mean age of women diagnosed with HCC was greater than that of men (65.1 vs. 61.3 years, *p* < 0.001). While the proportion of women with liver-associated comorbidities was less than that of men (36.1% vs. 42.3%, *p* = 0.012), more women were classified as obese (33.7% vs. 24.2%, *p* = 0.005). The proportion of women with early-stage disease (AJCC stage I, II) was greater than that of men (31.7% vs. 28.4%, *p* = 0.022). The proportion of patients who underwent any liver-directed surgery was greater among women than men (27.5% vs. 22%, *p* = 0.009), and the median time to treatment was shorter for women than for men (22 vs. 27 days, *p* = 0.001). No significant differences were noted in race, CCI, residence, or treatment location according to gender.

### 3.3. Racial Characteristics in Women

Table 2 summarizes cases of HCC by race. Of the 498 women with HCC, 188 (37.8%) were African American and 292 (58.6%) were white. The mean age of African American women was younger than that of White women (63.1 vs. 66.7 years, *p* = 0.001). The proportion of African American women living in a metropolitan area was greater than that of White women (91% vs. 84.6%, *p* = 0.004). The proportion of African American women treated in a high-volume parish was also marginally greater than that of White women (86.2% vs. 75%, *p* = 0.051). No significant differences were noted in CCI, liver-associated comorbidities, stage of disease, or receipt of surgical therapy among women based on race.

### 3.4. Women: Treatment and Survival

A comparison of patient characteristics according to surgical therapy is provided in Table 3; 137 (27.5%) women underwent liver-directed surgery while 361 (72.5%) did not. The mean age of women who underwent LDS was younger than those who did not (61.4 vs. 66.5 years, *p* < 0.001). The proportion of women with early-stage disease was significantly higher among those who underwent LDS (61.3% vs. 20.5%, *p* < 0.001), and the proportion of patients who were treated in a high-volume parish was greater among women who underwent LDS (94.9% vs. 74.2%, *p* < 0.001).

Of the 137 women who underwent LDS, 22 (16.1%) underwent ablation, 73 (53.3%) underwent resection, and 42 (30.7%) underwent transplantation. This was different, though not significantly, than what was observed in the 469 men who underwent LDS, where ablation, resection, and transplantation were utilized 17.7%, 43.3%, and 39% of the time, respectively (*p* = 0.105). The mean age of women undergoing resection or ablation was greater than those undergoing transplantation (*p* < 0.001). The proportion of women with early-stage disease was greater amongst those who underwent transplantation (*p* = 0.028).

**Figure 1:**
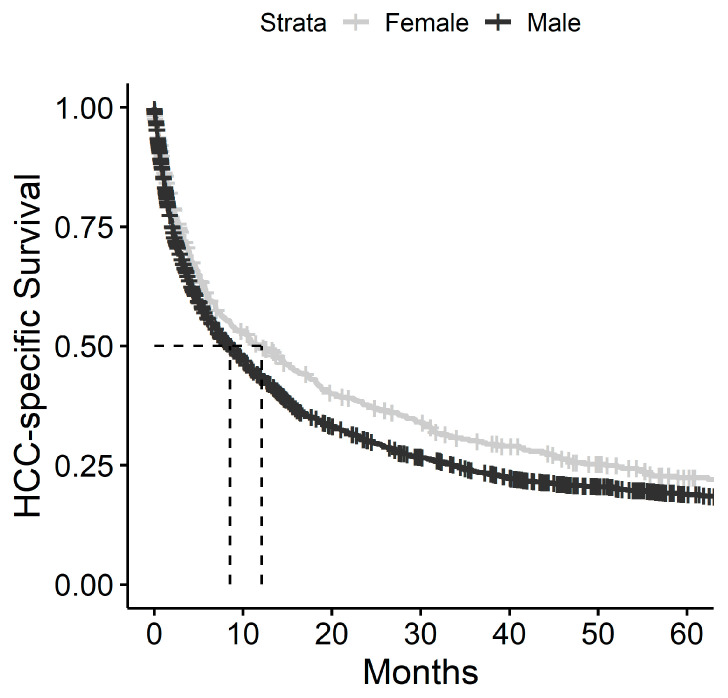
Kaplan–Meier cause-specific survival curves depicting cases of HCC in Louisiana, comparing Men vs. Women. Median survival times are indicated by the dashed lines.

**Figure 2:**
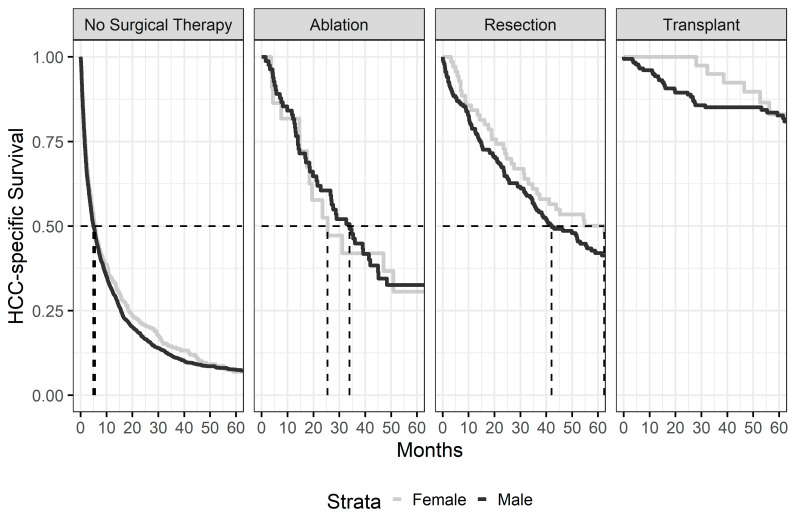
Kaplan–Meier survival curves comparing Men vs. Women, controlling for therapy received. Median survival times are indicated by the dashed lines.

**Figure 3:**
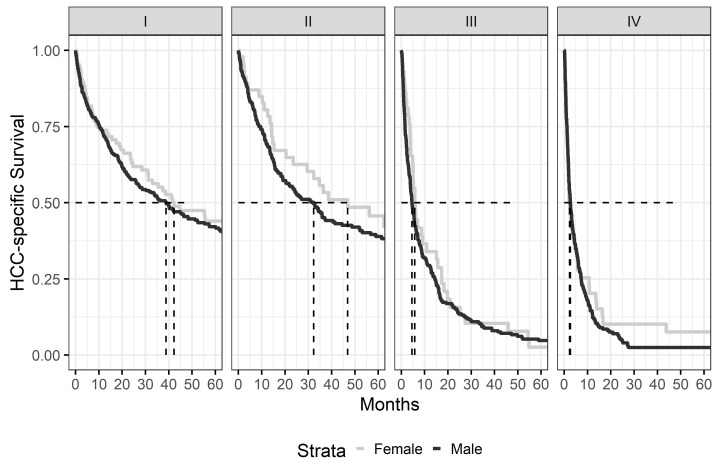
Kaplan–Meier survival curves comparing Men vs. Women controlling for stage of HCC at time of diagnosis. Median survival times are indicated by the dashed lines.

Kaplan–Meier survival curves according to gender are presented as Figure 1. Median HCC—specific survival (HSS) for the whole cohort was 9 months—12.1 months in women and 8.5 months in men (*p* = 0.005, Figure 1). When looking exclusively at patients who received LDS, there was no difference in HSS between men and women, and this finding was observed regardless of the type of LDS that was utilized (Figure 2). Additionally, no differences in HSS were observed between men and women based on the stage of the disease (Figure 3).

Results from the Cox proportional hazard models of HSS are given in Table 4. In Model 1, independent associations for worse HSS were male gender (*p* = 0.029), age greater than 65 years (*p* < 0.001), African American race (*p* < 0.001), late-stage (*p* < 0.001), and treatment in a low-volume parish (*p* < 0.001). However, in Model 2, which controlled for LDS, the lack of LDS was the strongest associated factor (HR (95% CI): 3.66 (3.19–4.21), *p* < 0.001), and gender was no longer predictive of worse HSS (HR (95% CI): 1.08 (0.96–1.21), *p* < 0.001). Factors predictive of worse HSS were similar between men and women, with the exception that women aged greater than 65 (HR (95% CI): 1.08 (0.87–1.34), *p* = 0.465) and African American race (HR (95% CI): 1.14 (0.91–1.41), *p* = 0.239) were not significantly associated with differences in HSS.

## 4. Discussion

Women comprise the minority of patients with HCC in the United States and around the world. Multiple explanations for this gender discrepancy have been suggested—some are based on gender-associated behavior patterns. When analyzing the HCC patient population, male patients consistently have higher rates of viral hepatitis, alcohol consumption, and concomitant hepatitis and alcohol use [5,6]. Recent studies have cited that up to 75% of HCC cases in male patients are caused by a combination of these two factors, compared to 51% of HCC cases in women [22]. Additionally, viral hepatitis and alcohol use are hypothesized to act synergistically in the development of HCC [11].

Other theories indicate that an underlying biological and hormonal difference between genders—such as estrogen’s interference in inflammatory signaling pathways—explains the delay in tumorigenesis in female hepatocytes [7,23,24]. Regardless, understanding gender disparities is critical to optimizing individual long-term cancer outcomes, and there are unique concerns that should be identified and highlighted when managing women with this malignancy. Well-known risk factors for HCC include hepatitis, heavy alcohol consumption, and obesity, in addition to low socioeconomic status. Louisiana is the top among states in the union for each of these risk factors and consequently ranks number two in the United States in HCC mortality, making it fertile ground in which to study HCC at a more granular level.

In the present study, women comprised 19% of the entire cohort. This percentage is lower than the 33% published in studies using SEER [25], but similar to those studies from academic institutional datasets [22]. The percentage of African Americans and “other” racial groups was similar in both women and in men. We found that women with HCC were older than men at diagnosis, but they were more likely to present with early-stage disease. This trend has been repeatedly demonstrated by many others in the literature as well [22,25,26]. Women with HCC were more likely to suffer from obesity and less likely to harbor liver-associated comorbidities than men. This finding has been supported by many others who have found more non-cirrhotic HCC in women [8,9,22,27,28] in addition to a growing number of women with NAFLD-related HCC. Indeed, the most common etiology of HCC in non-cirrhotic patients is NAFLD [27,28,29].

Louisiana’s racial distribution is unique: African Americans with HCC are more prevalent while Asians and Hispanics with HCC are less populous than in other regions of the US. To our knowledge, this is the first study to exclusively consider the outcomes of African American women with HCC. In the present study, African American women were younger than White women with HCC, and more African American women with HCC resided in an urban setting. Consequently, African American women were more likely than White women to have their cancer treatment in a high-volume parish. While African Americans are a uniquely marginalized group that has been negatively impacted by many barriers to cancer care [30,31,32,33], this urban proximity to high-volume locations seems to have had a protective effect on African American women in this study. This is supported by the finding that treatment in a high-volume parish was independently associated with improved HSS, and was 1.6 times more predictive in women than it was in men. We also believe that this explains why African American race was predictive of worse HSS among men, but was not predictive of survival in women.

Women were more likely to receive LDS then were men, and this is likely explained by the earlier stage of disease among women at presentation. However, women were less likely to be transplanted then men; this is likely explained by the lower incidence of liver-associated comorbidities (i.e., less cirrhosis) in women, which makes them more amenable to non-transplant resection. In a 2020 study of over 5000 patients with HCC, cirrhosis was diagnosed in 98% of transplant recipients and only 58% of patients who underwent resection [22].

Female sex has been inconsistently associated with mortality in HCC. A 2018 study from the University of Hawaii analyzed 307 women and 899 men with HCC from the Pacific basin. Although women had smaller tumor size at diagnosis, less vascular invasion, and were more likely to have HCC diagnosed through screening, no survival differences were noted between the genders [9]. Phipps et al. studied over 5000 patients with HCC from five separate American academic centers and found that women had improved 1-, 3-, and 5-year overall survival when compared with men. However, recurrence rates between men and women were similar, and men were noted to have a higher rate of cirrhosis than women [22]. Thus, it’s highly possible that the 4% increase in overall survival that was observed by these authors was related to additional liver-associated—but not cancer-related—deaths among men. In an Italian study of 1834 patients, women with HCC were found to have smaller tumors with less vascular invasion and improved overall survival when compared with men. However, these authors felt this difference was a result of lead time bias as women were more compliant with screening, and the survival difference was no longer observed when they exclusively compared only patients diagnosed by screening [34].

In the present study, HSS for the whole cohort was 9 months, which is comparable to previous studies reporting approximately 6–12-month median survival [35,36,37]. Men had a worse HSS than women in the multivariate analysis. However, these results appear to be a function of treatment bias. Patients undergoing any type of LDS had significantly improved median survival compared to patients with no LDS. Additionally, more women than men underwent LDS, which also suggests that the improved HSS seen in the female population is a function of LDS. Moreover, when the multivariate model was performed controlling for LDS (Table 4, Model 2), the survival difference was attenuated. When survival was examined using the Kaplan–Meyer model, looking only at those patients who underwent LDS, median HSS between men and women was similar regardless of treatment strategy (Figure 2). Thus, the difference in survival noted between men and women appears to be a function of the more advanced stage and higher number of liver-associated comorbidities found in men, resulting in fewer men receiving LDS. However, when men can undergo LDS, the survival differences appear to be obviated.

The retrospective nature of this population-based registry presents many potential limitations, including implicit biases in both treatment and patient selection. Data on non-surgical treatments were not reliably captured, pre-diagnosis follow-up to assess the impact of screening was not available, and there was no way to assess the menopausal status or estrogen exposure of women in this dataset. Given the impact of non-surgical treatments such as hepatic artery embolization, future analyses including other treatment methods would be an interesting area of study. Additional limitations include the under-reporting or incomplete reporting of some variables, such as liver disease, and the potential for the migration of patients in and out of the registry area. Finally, there has been work looking at antigenic heterogenicity that has provided insight into the biology of HCC that was not captured by the database. However, this study utilized a large, heterogenous dataset comprised of patients from rural and urban settings, who were treated in both community and academic practices, in a state whose disease mortality ranks number two in America. We believe that this allows for a certain amount of generalizability to other treatment areas that are likely under-represented in datasets produced by academic centers. Moreover, the ability to evaluate women of color with HCC is a strength of this study—inherent to Louisiana’s demographics—that has been under-reported in the scientific community heretofore.

## 5. Conclusions

In conclusion, this study supports the idea that risk factors for HCC in women are different than those in men, and that they show a stronger association with obesity and a weaker association with cirrhosis. Consideration should be given to these gender differences when considering HCC screening recommendations. Although the oncogenic triggers in females are different than those in men, the response to treatment seems very similar.

## Figures and Tables

**Table 1:** HCC case characteristics, Louisiana 2005–2015.

	All (*n* = 2627)		Female (*n* = 498)		Male (*n* = 2129)		
	%	*n*	%	*n*	%	*n*	*p*-Value
Age, years							<0.001
mean (std)	62.1	10.4	65.1	12.0	61.3	9.9	
Age, years							<0.001
20–64	64.6	1698	48.0	239	68.5	1459	
65 and over	35.4	929	52.0	259	31.5	670	
Race							0.710
White	57.0	1497	58.6	292	56.6	1205	
Black	39.2	1030	37.8	188	39.6	842	
Other	3.8	100	3.6	18	3.9	82	
Charlson Comorbidity Index							0.128
2–3	56.0	1471	60.0	299	55.1	1172	
4	27.0	710	24.3	121	27.7	589	
5 or greater	17.0	446	15.7	78	17.3	368	
Liver Disease							0.012
No	58.9	1546	63.9	318	57.7	1228	
Yes	41.2	1081	36.1	180	42.3	901	
Stage at Diagnosis							0.011
I	18.0	473	22.1	110	17.1	363	
II	11.0	289	9.6	48	11.3	241	
III	11.9	313	9.0	45	12.6	268	
IV	11.6	304	9.8	49	12.0	255	
Unknown	47.5	1248	49.4	246	47.1	1002	
Residence							0.524
Non-metropolitan	13.3	350	12.5	62	13.5	288	
Metropolitan	86.7	2277	87.6	436	86.5	1841	
Parish Treatment Volume							0.468
High	78.7	2068	79.9	398	78.4	1670	
Low	21.3	559	20.1	100	21.6	459	
Liver-directed Surgery							0.008
None	76.9	2021	72.5	361	78.0	1660	
Ablative	4.0	105	4.4	22	3.9	83	
Resection	10.5	276	14.7	73	9.5	203	
Transplant	8.6	225	8.4	42	8.6	183	

**Table 2:** HCC in women, case characteristics by race—Louisiana 2005–2015.

	All (*n* = 498)		White (*n* = 292)		Black (*n* = 188)		
	%	*n*	%	*n*	%	*n*	*p*-Value
Age, years							0.001
mean (std)	65.1	12.0	66.7	12.3	63.1	11.5	
Age, years							<0.001
20–64	48.0	239	40.1	117	58.0	109	
65 and older	52.0	259	59.9	175	42.0	79	
Charlson Comorbidity							0.284
2–3	60.0	299	57.2	167	63.8	120	
4	24.3	121	26.7	78	20.7	39	
5 or greater	15.7	78	16.1	47	15.4	29	
Liver Disease							0.244
No	63.9	318	61.6	180	67.0	126	
Yes	36.1	180	38.4	112	33.0	62	
Stage at Diagnosis							0.293
I	22.1	110	22.3	65	20.7	39	
II	9.6	48	8.6	25	11.2	21	
III	9.0	45	9.3	27	8.5	16	
IV	9.8	49	7.5	22	12.8	24	
Unknown	49.4	246	52.4	153	46.8	88	
Residence							0.004
Non-metropolitan	12.5	62	15.4	45	9.0	17	
Metropolitan	87.6	436	84.6	247	91.0	171	
Parish Treatment Volume							0.051
High	79.9	398	75.0	219	86.2	162	
Low	20.1	100	25.0	73	13.8	26	
Surgical Therapy							0.674
No	72.5	361	72.6	212	74.5	140	
Yes	27.5	137	27.4	80	25.5	48	

**Table 3:** HCC in women, case characteristics by surgical therapy—Louisiana 2005–2015.

			Liver-Directed Surgery				
	All (*n* = 498)		No (*n* = 361)		Yes (*n* = 137)		
	%	*n*	%	*n*	%	*n*	*p*-Value
Age, years							<0.001
mean (std)	65.1	12.0	66.5	12.0	61.4	11.3	
Age, years							<0.001
20–64	48.0	239	42.7	154	62.0	85	
65 and older	52.0	259	57.3	207	38.0	52	
Race							0.085
White	58.6	292	58.7	212	58.4	80	
Black	37.8	188	38.8	140	35.0	48	
Other	3.6	18	2.5	9	6.6	9	
Charlson Comorbidity							0.775
2–3	60.0	299	60.4	218	59.1	81	
4	24.3	121	24.7	89	23.4	32	
5 or greater	15.7	78	15.0	54	17.5	24	
Any Liver Disease							0.349
No	63.9	318	65.1	235	60.6	83	
Yes	36.1	180	34.9	126	39.4	54	
Stage at Diagnosis							<0.001
I	22.1	110	15.8	57	38.7	53	
II	9.6	48	4.7	17	22.6	31	
III	9.0	45	10.5	38	5.1	7	
IV	9.8	49	12.5	45	2.9	4	
Unknown	49.4	246	56.5	204	30.7	42	
Residence							0.774
Non-metropolitan	12.5	62	12.2	44	13.1	18	
Metropolitan	87.6	436	87.8	317	86.9	119	
Parish Treatment Volume							<0.001
High	79.9	398	74.2	268	94.9	130	
Low	20.1	100	25.8	93	5.1	7	

**Table 4:** Factors associated with HCC-specific survival from Cox proportional hazards models. Risk estimates are given as hazard ratios (HR) and 95% confidence intervals (CI). Model 1 evaluates the effect of age, sex, race, liver disease, stage, and parish case volume. Model 2 includes the previously mentioned covariates as well as LDS.

	Model 1		Model 2	
	HR (95% CI)	*p*-Value	HR (95% CI)	*p*-Value
Sex (ref = Female)		0.029		0.224
Male	1.14 (1.01, 1.28)		1.08 (0.96, 1.21)	
Age (ref = 20–64)		<0.001		0.013
65 and over	1.19 (1.09, 1.31)		1.13 (1.03, 1.24)	
Race (ref = White)		<0.001		0.002
Black	1.21 (1.10, 1.33)		1.14 (1.04, 1.25)	
Other	0.77 (0.60, 0.99)		0.80 (0.62, 1.02)	
Liver Disease (ref = No)		0.862		0.813
Yes	1.01 (0.92, 1.11)		0.99 (0.90, 1.09)	
Stage (ref = Early)		<0.001		<0.001
Late (Stage III and IV)	3.65 (3.20, 4.16)		2.52 (2.20, 2.88)	
Unknown	2.30 (2.05, 2.59)		1.73 (1.53, 1.95)	
Parish Treatment Volume (ref = High)		<0.001		<0.001
Low	1.70 (1.53, 1.89)		1.40 (1.26, 1.56)	
Liver-directed Surgery (ref = Yes)				<0.001
No			3.66 (3.19, 4.21)	

## Data Availability

Study data was derived from the Louisiana Tumor Registry (LTR). The LTR is a population-based registry funded by the NCI’s SEER Program and the Centers for Disease Control and Prevention’s National Program of Cancer Registries (NCPR). Data requests can be made at https://sph.lsuhsc.edu/louisiana-tumor-registry/data-usestatistics/data-request/ (accessed on 2 September 2020).
